# Depression and anxiety among people with hypertension on follow-up in Eastern Ethiopia: A multi-center cross-sectional study

**DOI:** 10.3389/fpsyt.2022.853551

**Published:** 2022-11-11

**Authors:** Lemesa Abdisa, Shiferaw Letta, Kabtamu Nigussie

**Affiliations:** ^1^Department of Nursing, School of Nursing and Midwifery, College of Health and Medical Science, Haramaya University, Harar, Ethiopia; ^2^Department of Psychiatry, School of Nursing and Midwifery, College of Health and Medical Sciences, Haramaya University, Harar, Ethiopia

**Keywords:** hypertension, depression, anxiety, magnitude, Eastern Ethiopia, follow-up

## Abstract

**Background:**

People with hypertension have a high risk of developing mental disorders, such as depression and/or anxiety. However, there is a paucity of data regarding comorbid depression and anxiety symptoms among people with hypertension in study settings.

**Objective:**

The study determined the prevalence and associated factors of depression, and anxiety symptoms among people with hypertension on follow-up at public hospitals, in Eastern Ethiopia.

**Materials and methods:**

A cross-sectional study was carried out among 471 people with hypertension who were randomly chosen from four public hospitals in Harar town and the Dire Dawa Administration. The data were collected by interviewer-administered structured questionnaires. A validated nine-item Patient Health Questionnaire and Generalized Anxiety Disorder scales were used to assess depression and anxiety symptoms, respectively. A logistic regression model was used to identify the association among depression, anxiety, and their predictors. An adjusted odds ratio and a 95% confidence interval were used to report the association. The statistical significance was set at a *p*-value of < 0.05.

**Results:**

Depression and anxiety symptoms were present in 27.2 and 32.7% of people with hypertension, respectively. Being women (AOR = 1.74, 1.09–2.78), having no formal education (AOR = 2.19, 1.19–4.81), presence of other medical illnesses (AOR = 2.23, 1.39–3.56), having a family history of depression (AOR = 2.01, 1.25–3.19), and poor social support (AOR = 2.80, 1.60–5.22) were statistically associated with depressive symptoms, whereas being women (AOR = 1.54, 1.01–2.35), widowed and divorced (AOR = 2.22, 1.41–3.52), presence of other medical illnesses (AOR = 1.64, 1.06–2.53), and poor social support (AOR = 3.54, 2.09–6.01) were statistically associated with anxiety symptoms.

**Conclusion:**

More than a quarter of people with hypertension reported symptoms of depression and anxiety. Findings demonstrated that being a woman, having an additional medical illness and having poor social support were statistically associated with depressive and anxiety symptoms. Regular screening, early detection, and offering the proper intervention should be on top priorities for healthcare professionals.

## Introduction

Non-communicable diseases (NCDs) accounted for 41 million population deaths worldwide. Among these deaths, 32.8 million were attributed to cardiovascular diseases, diabetes, cancers, and chronic respiratory diseases (1). About 80% of these NCD deaths were recorded in low- and middle-income countries (2).

Hypertension is one of the NCDs, which is defined as abnormally high arterial blood pressure. Hypertension is diagnosed when the systolic blood pressure readings on 2 different days are greater than 140 mmHg or more, and/or the diastolic blood pressure readings on both days are greater than 90 mmHg or more (3). Each year, hypertension causes approximately 7.5 million deaths (4). The systematic review study revealed that hypertension (HTN) remains stable in developed countries, whereas it is rising in middle- and low-income countries (5). Adults with hypertension account for roughly a quarter of the population, and by 2025, that figure will rise to roughly one-third (6).

People with hypertension are at an increased risk of mental health disorders, including depression and/or anxiety (7). Changes in appetite and weight, sleep and activity patterns, energy levels, feelings of guilt, difficulty thinking clearly and making decisions, and recurring thoughts of death or suicide are all symptoms that indicate depression (8, 9). Anxiety is defined as the existence of excessive worry about several events or activities on the majority of days, as well as somatic symptoms like muscle tension, irritability, difficulty sleeping, and restlessness (9, 10).

The co-occurrence and the impact of psychological and psychosocial issues related to HTN are challenging in diagnosis and management (7). Depression likely causes a 5.7% increase in the global burden of diseases by 2020 and become the leading cause of disability worldwide by the year 2030 (6). Depression comorbidity decreases the quality of life and increases the risk of myocardial infarction and stroke in people with hypertension (11). Anxiety influences medication adherence in people with hypertension and limits the feature treatment options worsens the prognosis and increases mortality (12). Several studies showed that depression was found in 40.1% (13) to 58% (1), while anxiety ranged from 28.5% (14, 15) to 42.3% (1) in people with hypertension. Moreover, the prevalence of comorbid depression in Africa accounted for 33.3% (16).

Different studies showed that biological and psychosocial factors were associated with comorbid depression and anxiety among people with hypertension. These included gender, physical activity, socioeconomic status (13), concern about medication and poor BP control (17), comorbid chronic illness (1), weight change, low income (18), older age, family history of depression (19) and being a woman (1), stressful life event, comorbid diabetic poor social support (14), weight change, and low income (18). Though comorbid depression and anxiety adversely affect health outcomes, there is limited information about the prevalence and determinants in people with hypertension in Ethiopia, particularly in Eastern Ethiopia. Therefore, this study aimed to determine the prevalence and associated factors of depression and anxiety among people with hypertension, as well as to make important recommendations that will likely improve future intervention programs.

## Materials and methods

### Study design and setting

An institutional-based cross-sectional study design was conducted in four public hospitals found in Harar and Dire Dawa Administration, namely Hiwot Fana Specialized University Hospital, Jugal Hospital, Dilchora Referral Hospital, and Sabian General Hospital from June 15 to July 15, 2021. All these hospitals provide both in-patient and out-patient services for the population of Eastern Ethiopia including the surrounding regions and zones. Around 3,848 people with hypertension receive their services each year (20, 21).

### Eligibility criteria

All adults aged ≥18 years people who had been clinically diagnosed with hypertension and who were on follow-up during the study period at selected hospitals were included in the study. People who were critically ill, unable to communicate, and patients currently on antidepressants and anxiolytic medication were excluded from the study.

### Sample size determination and sampling procedure

The sample size was calculated by using a single population proportion formula, considering the following statistical assumptions: *z* = the standard value of confidence level of alpha 95%, *d* = the margin of error between the sample and the population (0.04). For this study, *p* (the estimated proportion of anxiety) = 24.7%, which was the prevalence of anxiety symptoms among people with hypertension conducted in Hawassa University Comprehensive Specialized Hospital, Southern Ethiopia (19).


$$\text{n}=\frac{{\left({\text{Z}}_{\alpha /2}\right)}^{2}\text{P}(1-\text{P})}{{\text{d}}^{2}}\text{n}=\frac{{\left(1.96\right)}^{2}0.247(1-0.247)}{0.04^{2}}=447$$

Accordingly, adding 10% for non-the response rate gives the total calculated sample size is 491. The data from each public hospital indicated that 363, 216, 239, and 205 people with hypertension were on follow-up at Dilchora Referral Hospital, Sabian General Hospital, Hiwot Fana Specialized University Hospital, and Jugal Hospital, respectively. Next, they were proportionally allocated to the sample size. As per allocation, 174, 104, 115, and 98 patients were from Dilchora Referral Hospital, Sabian General Hospital, Hiwot Fana Specialized University Hospital, and Jugal Hospital, respectively. A systematic random sampling method was used to select study participants from the designated public hospitals. The first study participant was selected by a lottery method from each hospital independently, and the subsequent study participants were chosen for every two people.

### Data collection procedure and measurements

Data were collected using face-to-face interviews, and a review of the patient’s chart for other comorbid medical illnesses, duration of treatment, and the number of antihypertensive medications. The questionnaire contains four parts: sociodemographic, clinical, psychosocial, and substance-related characteristics of the patients that were adapted and modified after reviewing similar literature.

Depression was measured by the nine items of the Patient Health Questionnaire (PHQ-9), validated in Ethiopia with Cronbach’s alpha of 0.84 (22, 23). Scores for each item are 0, “not at all”; 1, “several days”; 2, “more than half days” to 3, “nearly every day” with a total score ranging from 0 to 27. The respondent who scored above or equal to 10 was considered as having depression (24). Anxiety was assessed by generalized anxiety disorder 7-items (GAD-7), which was validated in Ethiopia and its Cronbach’s alpha was 0.95 (25, 26). Scores for each item were 0, “not at all”; 1, “several days”; 2, “more than half days” to 3, “nearly every day” with a total score ranging from 0 to 21. The respondents who scored above or equal to 10 were considered as having anxiety disorder (25).

Social support was assessed by Oslo Social Support Scale containing three items (Oslo-3). It is a three item questionnaire, commonly used to assess social support and it has been used in several studies. The sum score scale ranged from 3 to 14, which had 3 categories: poor support 3–8, moderate support 9–11, and strong support 12–14 (27), and it was validated in Ethiopia (28). Substance-related factors were assessed by alcohol, smoking, and substance involvement screening test (ASSIST), which is a brief screening questionnaire developed and validated by the World Health Organization (WHO) to find out people’s use of psychoactive substances currently and a history of ever substance use (29). Regular physical activity was assessed by 2 items of days in the last 7 days in a week. Then, the responses were added up (range, 0–14). Participants who scored ≥8 were coded as adhering to the physical activity recommendations (30). The medication side effect was assessed by asking patients and review patient’s chart. Data were collected by eight trained B.Sc nurses and supervised by two M.Sc nurses. Coronavirus disease (COVID-19) prevention protocol was completely applied during data collection.

### Data quality control

Data collectors and supervisors were trained for 2 days on the data collection approaches of the study. The questionnaire was translated into the local language Amharic by an expert and back to translated into English by another person to check it for consistency. A pretest was conducted on 5% of the sample size at Haramaya General Hospital to see the applicability of the instruments and feedback was incorporated into the final tool to improve the quality. The result was not included in the result of this study found. Throughout the data collection period, supervision was carried out, and the completeness and consistency of the questionnaire were looked over daily.

### Statistical analysis

The data were coded, cleaned, and entered into Epi Data version 3.1 and then exported to SPSS (Statistical Package for Social Science) version 20 for analysis. Bivariate and multivariate logistic regression analyses were performed to identify factors associated with the outcome variable. All variables with a *p*-value less than 0.05 in bivariate analysis were entered into the multivariate logistic regression analysis. A *p*-value of less than 0.05 was considered statistically significant, and the adjusted odds ratio (AOR) with a 95% confidence interval (CI) was calculated. The Hosmer–Lemeshow goodness test showed model fitness.

### Ethical consideration

Ethical clearance was obtained from the Institutional Health Research Ethics Review Committee (IHRERC) of Haramaya University College of Health and Medical Sciences. A formal letter of permission and support was provided to all four public hospitals in which the study was conducted. Participants were informed of the study’s objective, procedures, and information confidentiality, as well as their right to withdraw and stop the interview at any time. A written informed consent was taken from each study participant before data collection began. Confidentiality was maintained at all levels of the study through anonymous data collection. During data collection, the COVID-19 prevention protocol was strictly kept.

## Results

### Sociodemographic characteristics of participants

A total of 471 participants were included in the study, making a response rate of 96%. The median age of respondents was 50 years, interquartile range (IQR, 40–75) with the age range of 18–90 years. Around half of them, 51.2% (241) and 57.1% (269) were men and married, respectively. More than half, 59.9% (282) of participants were living with family, 201 (42.7%) were Muslim by religion, and nearly two-thirds 67.7% (319) were urban residents as presented in Table 1.

**TABLE 1:** Demographic information of people with hypertension on follow-up in Eastern Ethiopia (*n* = 471).

Variables	Categories	Frequency (*n* = 471)	Percentage (%)
Sex	Men	241	51.2
	Women	230	48.8
Age category in year	18–39	92	19.5
	40–49	140	29.7
	50–59	124	26.3
	≥60	115	25.3
Marital status	Single	67	14.2
	Divorced/widowed	135	28.7
	Married	269	57.1
Living arrangement	Alone	189	40.1
	With family	282	59.9
Religion	Muslim	201	42.7
	Orthodox	171	36.3
	Protestant	70	14.9
	Others	29	6.2
Occupation	Framer	59	12.5
	Merchant	136	28.9
	Civil servant	180	38.2
	Household worker	77	16.3
	Jobless	19	4.0
Education	No formal education	58	12.3
	Primary school (1–8)	85	18.0
	Secondary school (9–12)	107	27.7
	College and above	221	46.9
Place residence	Urban	319	67.7
	Rural	152	32.3
Monthly income, ETB	≤1400	119	25.3
	1401–3500	124	26.3
	3501–5000	12999	27.421.0
	≥5000	99	21.0

NB: one Ethiopian Birr = 0.023 US dollar.

### Clinical, psychosocial, and substance-related factors of respondents

About two-thirds, 63.9% of participants were reported as having no family history of hypertension. Around half, 54.4% (256) of respondents had up to 5 years duration of treatment and 53.8% (254) of them were taking two antihypertensive medications, whereas more than half 55.6% (260) were with controlled hypertension. More than two-thirds, 67.7% (319) of participants with hypertension had no regular physical activities. From all study respondents, 61.8% (291) and 36.7% (173) had 18.5–24.9 kg/m^2^ body mass index and moderate social support, respectively, whereas 42.5% (200) had a history of other comorbid medical illnesses. Concerning substance use, nearly one-third 34.8% (164) of them smoke a cigarette as presented in Table 2.

**TABLE 2:** Clinical, substance use, and psychosocial characteristics of people with hypertension on follow-up in Eastern Ethiopia (*n* = 471).

Variables	Categories	Frequency (*n* = 471)	Percentage
Family history of hypertension	Yes	170	36.1
	No	301	63.9
Duration of treatment	Up to 5 years	256	54.4
	5–10 years	146	31.4
	≥10 years	67	14.2
Family history of depression	Yes	180	38.2
	No	291	61.8
Number antihypertensive medication	One	175	37.2
	Two	254	53.8
	Three	42	8.9
Hypertension status	Controlled	260	55.2
	Uncontrolled	211	44.8
Comorbid medical illness	Yes	200	42.5
	No	271	57.5
Medication side effect	Yes	201	42.7
	No	270	57.3
Regular physical activity	Yes (regular)	152	32.3
	No (irregular)	319	67.7
BMI	<18.5	65	13.8
	18.5–24.9	291	61.8
	25–29.9	91	19.3
	≥30	24	5.1
Alcohol use	Yes	139	29.5
	No	332	70.5
Smoking	Yes	164	34.8
	No	307	65.2
Social support	Poor	135	28.7
	Moderate	173	36.7
	Strong	163	34.6

### Factors associated with depression symptoms in people with hypertension

In multivariable logistic regression analysis, variables, such as being a woman, not attending formal education, comorbid medical illness, family history of depression, and poor social support were statistically significantly associated with depression symptoms. In this study, the odds of having depression among respondents with being women was about 1.7 times higher compared with participants those being men (AOR = 1.74; 95% CI: 1.09–2.78), and the odds of having depression among participants who did not attend formal education was 2.2 times higher compared with the respondents who attended college and above education (AOR = 2.19; 95% CI: 1.19–4.81). The odds of having depression among respondents who had other medical illnesses was 2.23 times higher compared with the respondents who had no other medical illness (AOR = 2.23; 95% CI: 39–3.56) and odds of having depression among participants who had a family history of depression was 2.0 times higher compared with the participants those who had no family history of depression (AOR = 2.01; 95% CI: 1.25–3.19). The finding of this study indicated that the odds of having depression among respondents who had poor social support was about 2.8 times (AOR = 2.80; 95% CI: 1.60–5.22) higher compared with the participants who had strong social support as presented in Table 3.

**TABLE 3:** Factors associated with depressive symptoms in people with hypertension on follow-up in Eastern Ethiopia (*n* = 471).

Explanatory variables	Depression		COR (95% CI)	AOR (95% CI)
	
	Yes	No		
**Sex**				
Men	52	189	1	1
Women	76	154	1.79 (1.19–2.71)	1.74 (1.09–2.78)*
**Marital status**				
Single	24	43	1.86 (1.05–2.35)	1.71 (0.91–3.24)
Divorced/widowed	42	93	1.51 (0.95–2.39)	1.27 (0.75–2.13)
Married	62	207	1	1
**Educational status**				
No formal education	24	34	2.29 (1.25–4.21)	2.19 (1.19–4.81)*
Primary school	26	59	1.43 (0.82–2.49)	1.25 (0.67–2.33)
Secondary school	26	81	1.04 (0.61–1.79)	0.79 (0.43–1.48)
College and above	52	169	1	1
**Hypertension status**				
Uncontrolled	75	136	2.15 (1.43–3.26)	1.45 (0.88–2.41)
Controlled	53	270	1	1
**Comorbid illness**				
Yes	77	123	2.70 (1.78–4.09)	2.23 (1.39–3.56)**
No	51	220	1	1
**Body mass index in (kg/m^2^)**				
<18.5	11	54	1	1
18.5–24.9	77	214	1.77 (0.88–3.55)	1.49 (0.69–3.18)
25–29.9	34	57	2.93 (1.35–6.36	2.21 (0.93–5.28)
≥30	6	18	1.64 (0.53–5.06)	1.79 (0.44–1.14)
**Family history of depression**				
Yes	67	113	2.24 (1.48–3.38)	2.01 (1.25–3.19)*
No	61	230	1	1
**Smoking**				
Yes	54	110	1.55 (1.02–2.35)	0.71 (0.44–1.14)
No	74	233	1	1
**Social support**				
Poor	51	84	2.81 (1.65–4.77)	2.80 (1.60–5.22)**
Moderate	48	125	1.77 (1.05–2.99)	1.73 (0.97–3.09)
Strong	29	134	1	1

*p < 0.05, **p < 0.001, Chi square = 11.80; DF = 8 and Hosmer-Lemeshow test = 0.46.

### Factors associated with anxiety symptoms in people with hypertension

In this study, variables, such as being a woman, widowed/divorced, having a comorbid medical illness and having poor social support were significantly associated with anxiety symptoms. The odds of having anxiety among woman participants were about 1.5 times higher as compared with men participants (AOR = 1.54; 95% CI: 1.01–2.35), and the odds of having anxiety among divorced/widowed participants were 2.2 times higher as compared with married participants (AOR = 2.22; 95% CI: 1.41–3.52). The finding also indicated that the odds of having anxiety among participants who had comorbid other medical illnesses were about 1.6 times higher as compared with participants who had no comorbid other medical illness (AOR = 1.64; 95% CI: 1.06–2.53) and the odds of having anxiety among respondents who had poor social support was 3.5 times higher as compared with participants who had good social support (AOR = 3.54; 95% CI: 2.09–6.01) (Table 4).

**TABLE 4:** Factors associated with anxiety symptoms in people with hypertension on follow-up in Eastern Ethiopia (*n* = 471).

Explanatory variables	Anxiety		COR (95% CI)	AOR (95% CI)
	
	Yes	No		
**Sex**				
Men	66	175	1	1
Women	88	142	1.64 (1.11–2.42)	1.54 (1.01–2.35)*
**Marital status,**				
Single	17	50	0.89 (0.49–1.65)	0.79 (0.42–1.50)
Divorced/widowed	63	72	2.31 (1.49–3.55)	2.22 (1.41–3.52)**
Married	74	195	1	1
**Hypertension status**				
Uncontrolled	85	126	1.87 (1.27–2.76)	1.46 (0.95–2.27)
Controlled	69	191	1	1
**Comorbid illness**				
Yes	81	119	1.85 (1.25–2.73)	1.64 (1.06–2.53)**
No	73	198	1	1
**Social support**				
Poor	69	66	3.66 (2.16–5.86)	3.54 (2.09–6.01)**
Moderate	48	125	1.31 (0.79–2.14)	1.39 (0.84–2.34)
Strong	37	126	1	1

*p < 0.05, **p < 0.001, Chi square = 6.05; DF = 8 and Hosmer-Lemeshow test = 0.79.

## Discussion

This study indicated that the prevalence of comorbid depression among people with hypertension was 27.2% (95% CI: 22.9–31.2). Being a woman, having other medical illnesses, a family history of depression, and having poor social support showed the association with comorbid depression. Similarly, the prevalence of anxiety in the current finding was 32.7 % (95% CI: 28.2.3–36.9). Being a woman, divorced and widowed, having other medical illnesses, and having poor social support were predictors of comorbid anxiety in people with hypertension.

The prevalence of depressive symptoms in the current study was high, and this finding was in line with the study conducted in Nigeria (31), Pokhara Metropolitan City (32), and Hawassa, Ethiopia (19). However, it was lower than the study conducted in Saudi Arabia (19) and Nepal (33). The possible reason for the discrepancy might be the study design, as this study was an institutional-based cross-sectional study, whereas a study conducted in Saudi Arabia was a community-based cross-sectional study. In addition, data collection instruments and the economic status of the population might contribute to this variation. On the other hand, the finding of the current study was higher than the study conducted in Saudi Arabia (34), Korea (35), Shenzhen, China (36), and Northwest Ethiopia (37). The reason for the disparity might be the instrument used: the Beck Depression Inventory and the World Health Organization’s five wellbeing index were used in Saudi Arabia and Shenzhen, China, respectively, whereas the patient health questionnaire was used in this study.

The prevalence of anxiety symptoms in the current finding was high which was in line with the study conducted in China, Iran (38), and Addis Ababa, Ethiopia (14). However, the finding of the current study was higher than the previous studies conducted in Egypt (39), South Africa (40), Qatar (38), Germany (41), and Malaysia (42). The possible reason for the discrepancy might be sample size, study design, and study setting, a prospective cohort study and community-based survey were used in Germany (41) and Egypt (39), while this study used an institutional-based cross-sectional study design. Another possible reason for the difference might be the data collection instruments used, which were a Composite International Diagnostic Interview to measure DSM-IV mental disorders (40) and the Hospital Anxiety and Depression Scale (HADS) used in Malaysia (42). On the other hand, the result of the current study was lower than the study conducted in two hospitals in Ghana (42, 43). The possible reason for the discrepancy might be the difference in study participants, study design, and data collection tools, which were the Depression Anxiety Stress Scale (DASS) (42), Zung Self-Rating Anxiety Scale test (43), and Generalized Anxiety Disorder (GAD-7).

It was identified that poor social support was associated with depression and anxiety symptoms. Previous research conducted in Ethiopia provided evidence to support these findings (14, 19). This might be because the perceived feeling of being unsupported (isolated) and having a somatic illness (like hypertension) leads to increased psychosocial stress, and on the contrary, good social support reduces the risk of depression and anxiety (44).

In this study, having no formal education was associated with depression symptoms. This result was supported by the studies conducted in China (36), Saudi Arabia (34), and Ethiopia (19). The argument could be that people who had no formal education might have limited awareness about disease and poor coping mechanisms toward stress and psychosocial problems.

Having a family history of depression was correlated with depression symptoms. This might be that if one parent has a mood disorder (such as depression), the child has a risk of developing it (8) since they share similar life and psychosocial stress (19).

Being a woman was associated with depression and anxiety symptoms. This was supported by a study conducted in Karachi, Pakistan (45). The possible justification could be women are more vulnerable to psychosocial related problems due to hormonal effects (8). Those respondents, who had divorced/widowed higher risk to have anxiety because when they feel lonely, lack hope, are worthless and become anxious.

Other comorbid medical illnesses were associated with depression symptoms. This result was consistent with the number of studies conducted in different countries like Egypt (39), Pakistan (45), and central Ethiopia (14, 19). It is possible that metabolic factors, such as fasting blood glucose and systolic blood pressure, independently contribute to anxiety and depression (45), in addition to the direct pathophysiological effect of inflammation and metabolic factors on the hypothalamic-pituitary axis and autonomic nervous systems (46).

### Limitations of the study

The use of retrospective elements in the questionnaire may have incurred recall bias like duration of illness and duration of treatment. Because of the cross-sectional nature of the study and the lack of a control group, determining the cause–effect relationship between the outcome variable and predictive variables was difficult. The COVID-19 pandemic might also affect the result of this study.

### Future directions

With the existing study limitations, we recommended that researchers in the field of mental health and public health need to conduct an additional study about the prevalence of depression and anxiety through longitudinal studies and investigate the relationship between medication adherence and the grade of hypertension.

### Clinical and research implications of the study

This study might be an essential contribution in giving insight to the healthcare providers to evaluate the people with hypertension in the study area for psychiatric disorders and improve mental health services. The findings of this study will further motivate researchers to conduct the longitudinal study.

## Conclusion

The finding of this showed that depressive and anxiety symptoms were common among people with hypertension. Being a woman, having a family history of depression, having no formal education, having an additional medical illness, and having poor social support were significantly associated with depression. Likewise, being women, divorced/widowed, having other comorbid medical illnesses, and having poor social support were significantly associated with anxiety symptoms. Regular screening, early identification, diagnosis, and providing appropriate intervention might reduce adverse health outcomes resulting from depression and anxiety through a collaborative care model. In addition, an appropriate and timely referral from primary care to specialist care is recommended. Moreover, people with hypertension who were women, had an additional medical illness and poor social support need special attention.

## Data availability statement

The original contributions presented in the study are included in the article/supplementary material, further inquiries can be directed to the corresponding author.

## Ethics statement

The studies involving human participants were reviewed and approved by the Research Ethics Review Committee of College of Health and Medical Sciences, Haramaya University. The patients/participants provided their written informed consent to participate in this study.

## Author contributions

LA conceived the research idea. KN was involved from inception to design, acquisition of data, analysis, interpretation, and drafting and editing of the manuscript. LA and SL were involved in the reviewing of the proposal, analysis, interpretation, and critical review of the drafted manuscript. All authors read and approved the final manuscript.
